# Antioxidant Role of PRGF on RPE Cells after Blue Light Insult as a Therapy for Neurodegenerative Diseases

**DOI:** 10.3390/ijms21031021

**Published:** 2020-02-04

**Authors:** Carlota Suárez-Barrio, Susana del Olmo-Aguado, Eva García-Pérez, María de la Fuente, Francisco Muruzabal, Eduardo Anitua, Begoña Baamonde-Arbaiza, Luis Fernández-Vega-Cueto, Luis Fernández-Vega, Jesús Merayo-Lloves

**Affiliations:** 1 Instituto Universitario Fernández-Vega. Fundación de Investigación Oftalmológica & Universidad de Oviedo, 33012 Oviedo, Spain; 2 Instituto de Investigación Sanitaria del Principado de Asturias, Avenida de Roma s/n., 33011 Oviedo, Spain; 3 BTI Biotechnology Institute, 01007 Vitoria, Spain; 4 University Institute for Regenerative Medicine and Oral Implantology—UIRMI (UPV/EHU—Fundación Eduardo Anitua), 01007 Vitoria, Spain

**Keywords:** age-related macular degeneration (AMD), neurodegenerative disease, retinal pigment epithelium (RPE), ARPE19, plasma rich in growth factors (PRGF), blue light, reactive oxygen species (ROS), antioxidant

## Abstract

Oxidative stress has a strong impact on the development of retinal diseases such as age-related macular degeneration (AMD). Plasma rich in growth factors (PRGF) is a novel therapeutic approach in ophthalmological pathologies. The aim of this study was to analyze the antioxidant effect of PRGF in retinal epithelial cells (EPR) in in vitro and ex vivo retinal phototoxicity models. In vitro analyses were performed on ARPE19 human cell line. Viability and mitochondrial status were assessed in order to test the primary effects of PRGF. GSH level, and protein and gene expression of the main antioxidant pathway (Keap1, Nrf2, GCL, HO-1, and NQO1) were also studied. Ex vivo analyses were performed on rat RPE, and HO-1 and Nrf2 gene and protein expression were evaluated. The results show that PRGF reduces light insult by stimulating the cell response against oxidative damage and modulates the antioxidant pathway. We conclude that PRGF’s protective effect could prove useful as a new therapy for treating neurodegenerative disorders such as AMD.

## 1. Introduction

Age-related macular degeneration (AMD) is a neurodegenerative disease that leads to deficiencies of retinal pigment epithelium (RPE) and, consequently, reduction in visual function [1]. The RPE is a single layer of cells adjacent to the outer segment of photoreceptor of the retina and Bruch’s membrane [2]. Among the main functions of RPE are the maintenance and survival of photoreceptors, which it achieves by providing nutrients taken from the choroidal capillaries, as well as the protection of cones and rods from free radicals [3]. The macula corresponds to the portion of the RPE that creates defined images. As such, it is involved in face recognition, reading, and other activities that require sharp definition vision, and for this reason maintenance of the integrity of the macular is of great importance [4]. The causes of AMD are not fully understood, although the aging process and oxidation caused by the presence of reactive oxygen species (ROS) are known to be the principal factors behind this pathology [5,6,7].

RPE cells, such as rods and cones, are rich in mitochondria. In this regard, the importance of the activity of this organelle to produce energy for the maintenance of cell function is well known [8]. This energy takes the form of adenosine triphosphate (ATP) and it is produced by the electron flow carried out by the four complexes of the electron transport chain, which is governed by fundamental enzymes in the mitochondrial inner membrane [9,10]. Nevertheless, the presence of ROS can counteract the enzymes’ functions by interrupting their reactions, thus, reducing ATP production and, as a result, leading to oxidative stress and cell death [11,12].

ROS output can be increased by a number of factors, including the aging process, smoking, and obesity, all of which contribute to the detriment of RPE cells [13]. However, there is one other factor, the incidence of which has increased in importance considerably in recent years due to its widespread introduction in contemporary life [14]. This factor is the influence of blue light, i.e. short wavelength light of 400 to 500 nm [12]. This wavelength corresponds with the visible spectrum that reaches the retina and is one of the main fractions of new white illumination [15,16]. The constant exposure of people in modern developed society to this type of light results in their increased risk of suffering from a variety of ocular disorders [16,17,18,19,20,21]. Moreover, there is some evidence that the action of blue light on the eye is increased when tissues are damaged or in suboptimal conditions, thus, worsening its impact on individuals with pathological conditions [22,23,24,25,26,27].

In order to address this situation, researchers have been trying to find a solution to reduce this harm through the use of antioxidants. Expression of these molecules is stimulated by the increased releasing of ROS in order to counteract oxidant species and block their action. Hence, they are able to reduce the damage caused by oxidation.

There is some evidence that suggests plasma rich in growth factors (PRGF) could act as a protector [28]. PRGF is a serum that is extracted from a patient’s blood. The procedure to obtain it involves removing the white cell series from blood, and therefore avoids the natural immunological response. Its beneficial effect has been proven with respect to various pathologies. It has been widely used in oral implantology and traumatology in order to enhance wound healing [29,30,31]. With this same purpose, it has been used in ophthalmology to treat ocular surface diseases such as persistent epithelium defects (PED) and other pathologies [32,33,34,35,36]. It contributes to the acceleration of the healing process by increasing the proliferation and migration rate of cells. In addition, it is also involved in eye hydration [37] and reducing inflammation. Previous research has shown the retina responds well to PRGF treatment [38].

Therefore, the inherent features of PRGF suggest that it could act as an antioxidant agent. Consequently, we studied one the of the most important antioxidant pathways, the Keap1-Nrf2 pathway, in order to test whether PRGF enhances the expression of antioxidant molecules. Kelch-like ECH-associated protein 1 (Keap1) binds to nuclear factor erythroid-related factor 2 (Nrf2) in the cytoplasm, keeping it inactivated. When ROS production increases, specific cysteine residues of Keap-1 are modified, releasing Nrf2 [39]. This translocates to nucleus, activating the expression of antioxidant enzymes such as heme oxygenase-1 (HO-1), glutamate-cysteine ligase (GCL), and NAD(P)H:quinone oxidoreductase (NQO1). These molecules join to ROS, blocking their action and reducing their effect, and thus protect cells against oxidation [40].

## 2. Results

### 2.1. In Vitro Viability Assays in ARPE19 Cell Cultures

Cell viability was measured at 1 + 18 h on ARPE19 cell cultures under the following treatments (see Table 1). For this, we performed MTT assays (*n* = 24) to assess the viability differences based on the treatment applied.

**Figure 1:**
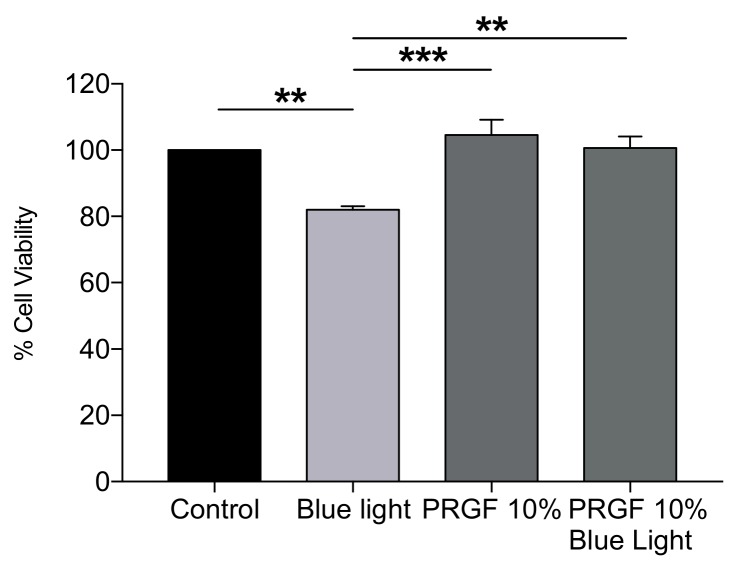
ARPE19 MTT assay (*n* = 24) for viability. Plasma rich in growth factors (PRGF) 10% protects cells against blue light insult by increasing cell viability up to control levels. Statistical analysis: One-way Anova, Tukey multiple comparison test, ** *p* < 0.005, and *** *p* < 0.0005.

After 19 h of experimental conditions, the results demonstrated that blue light reduced cell viability by around 20% as compared with the control treatment. We also found that PRGF did not significantly alter cell viability as compared with the control, indicating that PRGF is not in itself toxic to cell. However, the most important result we found was that cell viability recovered to a statistically significant extent when blue light was combined with PRGF 10%, with it reaching normal values as compared with the control (Figure 1).

### 2.2. Analysis of ARPE19 Mitochondria Status and ROS Production

JC-1 staining was used to test mitochondrial status. This dye has two forms. It is red when mitochondria are in a healthy condition and green when they are depolarized. For this aspect of the trial, ARPE19 cells were divided into the same four groups previously described.

**Figure 2:**
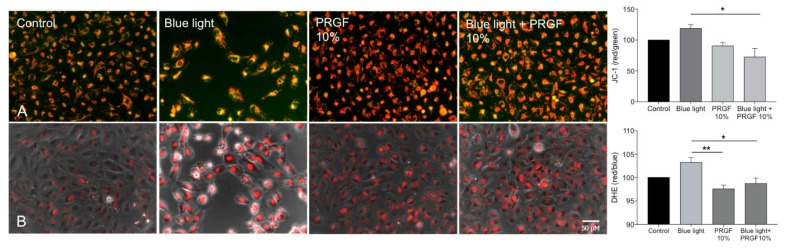
ARPE19 cell cultures (*n* = 4). (**A**) JC-1 staining to study mitochondria status, PRGF enhances mitochondria status in presence of blue light; (**B**) Dihydroethidium staining (DHE) for the presence of reactive oxygen species (ROS), PRGF significantly reduces the expression of ROS as compared with the blue light treatment. Statistical analysis: One-way ANOVA, Tukey multiple comparison test, * *p* < 0.05, and ** *p* < 0.005. Scale = 50 µM.

Cells exposed to blue light showed more depolarization than those in the control condition, suggesting that the mitochondria could be in a worse condition. In addition, PRGF 10% and PRGF 10% + blue light groups showed more polarization than the control. Quantitative analysis showed that PRGF significantly reduced the effect of blue light in cell mitochondria (Figure 2A). 

Dihydroethidium staining (DHE) was used to check for any changes in ROS production in ARPE19 cells that were dependent on the treatment. Analysis showed that blue light increased the presence of ROS, while PRGF 10% reduced it significantly in both treatment scenarios where it was used (Figure 2B).

### 2.3. GSH Quantification and GCL Gene and Protein Expression

**Figure 3:**
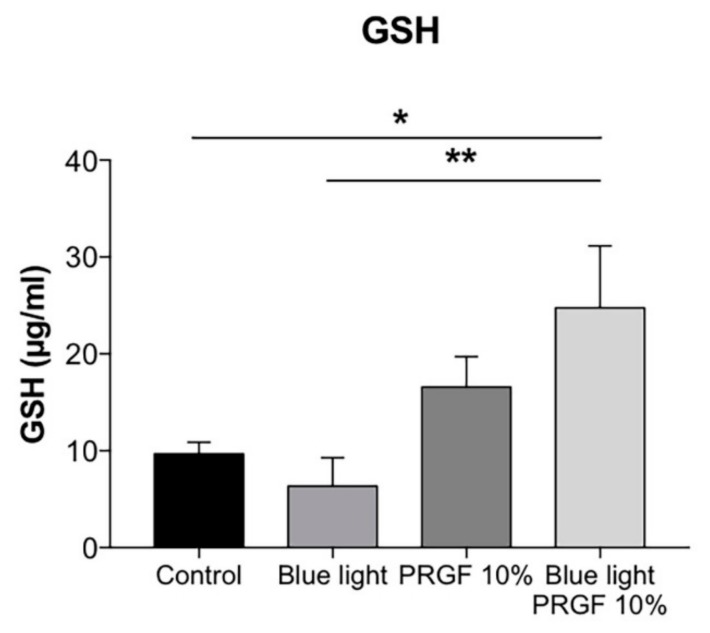
GSH quantification in ARPE19 cultures (*n* = 4). Blue light reduced the concentration of GSH, while PRGF significantly increased GSH compared to control. Statistical analysis: One-way ANOVA, Tukey multiple comparison test, * *p* < 0.05, and ** *p* < 0.005.

A glutathione assay kit was used to quantify the concentration of GSH (the reduced form of glutathione) in ARPE19 cell cultures. Cells were divided into the same four treatment groups described above. The results showed that blue light reduced GSH levels, although the difference was not significant as compared with the control (Figure 3).

The increase in ROS due to blue light could cause this result. Therefore, GSH is oxidized in order to protect cells against oxidant molecules. However, PRGF, whether in combination with blue light or not, significantly increased the expression of GSH.

**Figure 4:**
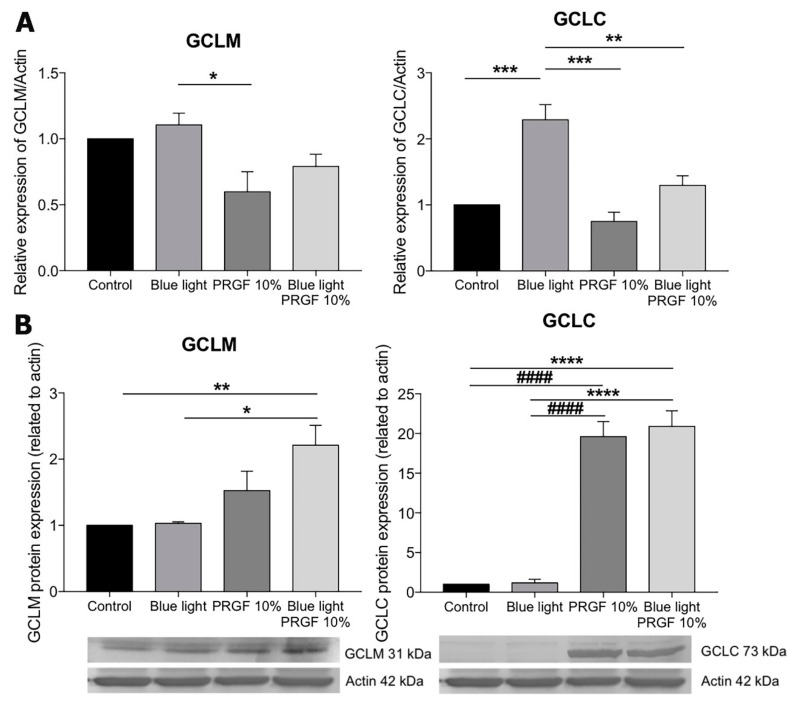
(**A**) GLCM and GCLC gene expression related to actin in ARPE19 (*n* = 4), PRGF reduced their expression in both the presence and absence of blue light; (**B**) GCLM and GCLC Western blot analysis in ARPE19, PRGF increased their expression in both cases. Statistical analysis: One-way ANOVA, Tukey multiple comparison test, * *p* < 0.05, ** *p* < 0.005, *** *p* < 0.0005, **** *p* < 0.0001, and ^####^ *p* < 0.0001.

In order to test the GSH pathway, we studied the expression of GCLM and GCLC. The results of qPCR showed that the expression of both glutamate-cystein ligase (GCL) subunits was increased by the presence of blue light (Figure 4A).

However, the increase was only significant for GCLC. The presence of PRGF, on the one hand, resulted in the significantly reduced expression of both molecules as compared with the blue light group, on the other hand for GCLC, the effect was significant whether or not PRGF was combined with blue light. Western blot analysis showed that PRGF increased the levels of both subunits in both the presence and absence of blue light as compared with the control group (Figure 4B).

**Figure 5:**
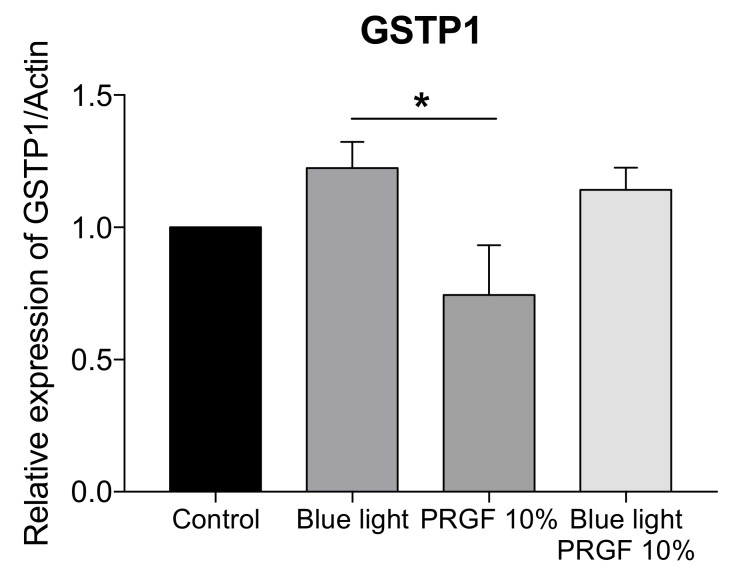
Gene expression of GSTP1 related to actin in ARPE19 cell cultures (*n* = 4). Blue light increased the expression of GSTP1, and PRGF reduced it significantly. Statistical analysis: One-way ANOVA, Tukey multiple comparison test, and * *p* < 0.05.

Tests for the gene expression of GSTP1 were also performed. This molecule modulates the conjugation of GSH with hydrophobic and electrophilic compounds, protecting cells against ROS. The results (Figure 5) showed that blue light induced an increase in GSTP1 expression while PRGF reduced it significantly.

### 2.4. Keap1-Nrf2 Antioxidant Pathway

**Figure 6:**
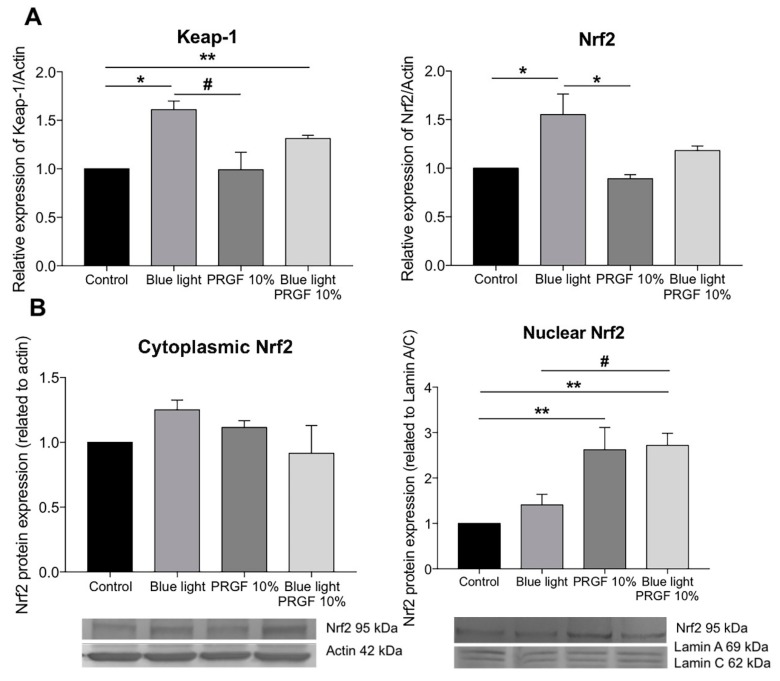
ARPE19 cell cultures (*n* = 4). (**A**) Nrf2 and Keap1 gene expression related to actin, blue light increased the expression of both genes but PRGF reduced it; (**B**) Nrf2 protein expression, blue light slightly increased the expression of cytoplasmic Nrf2 as compared with the control, nuclear Nrf2 increased when cells were treated with PRGF. Statistical analysis: One-way ANOVA, Tukey multiple comparison test, * *p* < 0.05, ** *p* < 0.005, and # *p* < 0.05.

We also studied the response of the Keap1-Nrf2 pathway to blue light and PRGF. The molecular results showed that blue light significantly increased the gene expression of both Nrf2 and Keap1 as compared with both the control group and PRGF 10% (Figure 6A). However, the expression of Keap1 in the blue light alone group was significantly different to the PRGF combined with blue light group. In terms of protein expression, we analyzed Nrf2 expression in cytoplasm and nucleus (Figure 6B). Cytoplasmic Nrf2 expression was increased in the presence of blue light but this was not statistically significant. However, nuclear Nrf2 was higher in the presence of PRGF as compared with the control. The results, therefore, show that PRGF increased the expression of antioxidant molecules.

To complete our analysis of the antioxidant pathway, we also studied the gene expression of molecules such as HO-1 and NQO1.

**Figure 7:**
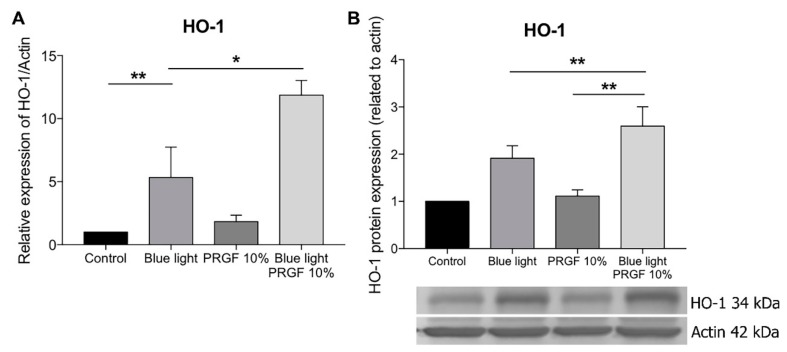
ARPE19 cell cultures. (**A**) HO-1 gene expression related to actin, blue light stimulated the expression of HO-1 as compared with the control, PRGF combined with blue light treatment showed a higher expression of HO-1 as compared with the other groups; (**B**) HO-1 protein expression related to actin, blue light increased the expression of HO-1 as did PRGF combined with blue light treatment. Statistical analysis: One-way ANOVA, Tukey multiple comparison test, * *p* < 0.05, and ** *p* < 0.005.

**Figure 8:**
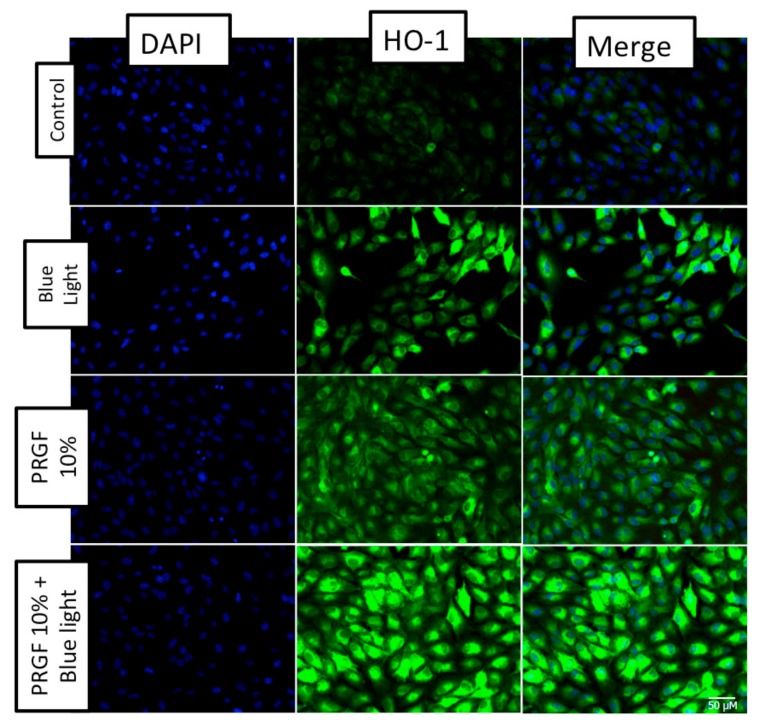
ARPE19 immunocytochemistry for HO-1 (green) and DAPI (blue) (*n* = 4). The results showed that blue light increased HO-1 expression. In addition, PRGF intrinsically expressed HO-1 as compared with the control. PRGF combined with blue light also increased staining for HO-1. Scale = 50 µM.

We found that HO-1 gene expression was stimulated by the presence of blue light (Figure 7A), as well as in the group where PRGF was combined with blue light. The Western blot (Figure 7B) and immunocytochemistry (Figure 8) results for HO-1 confirmed that PRGF stimulated the expression of HO-1 in the presence of blue light.

**Figure 9:**
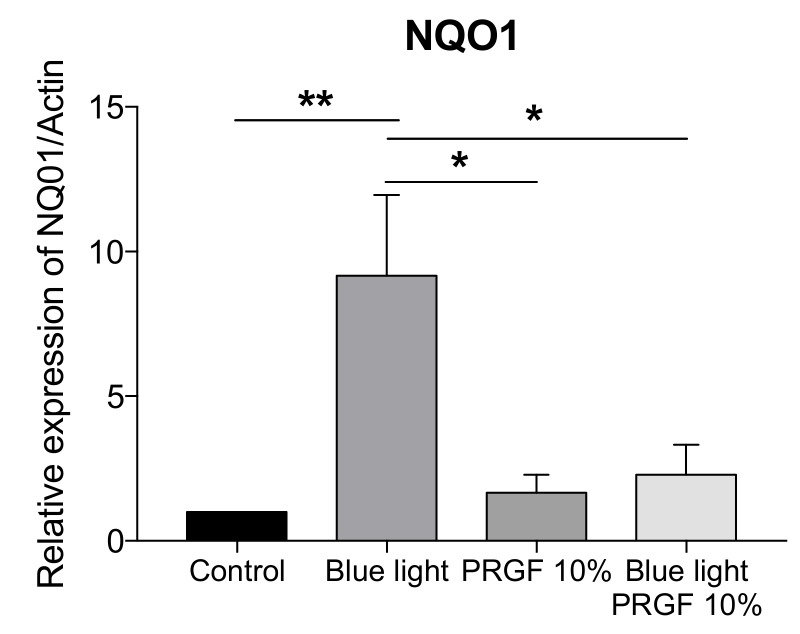
ARPE19 qPCR for NQO1 related to actin (*n* = 4). NQO1 expression is increased by blue light. However, PRGF reduced it significantly. Statistical analysis: One-way ANOVA, Tukey multiple comparison test, * *p* < 0.05, and ** *p* < 0.005.

The molecular analysis of the NQO1 gene showed that blue light significantly increased its expression, however, PRGF, in combination with blue light or not, reduced it (Figure 9).

### 2.5. RPE Antioxidant Analysis in an Ex Vivo Model

An ex vivo model was performed to study the antioxidant effect on RPE of HO-1 and Nrf2. For this, rat eyecups were exposed to various conditions in order to observe the response of the tissue to the different insults (see Table 2).

**Figure 10:**
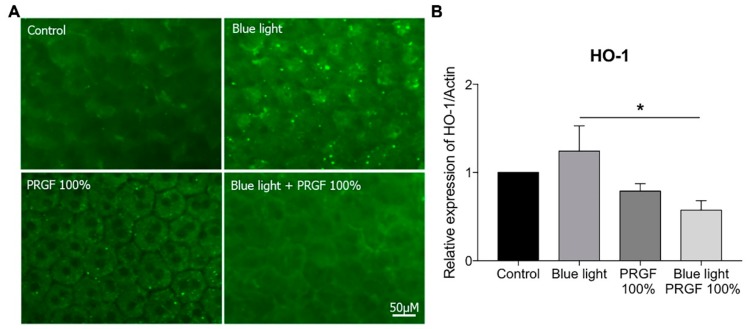
(**A**) RPE immunocytochemistry for HO-1 (green) (*n* = 4), results showed that blue light increased HO-1 expression but PRGF 100% reduced it; (**B**) expression of HO-1 is increased by blue light, PRGF 100% significantly reduced its expression when combined with blue light. Statistical analysis: One-way ANOVA, Tukey multiple comparison test, and * *p* < 0.05. Scale = 50 µM.

**Figure 11:**
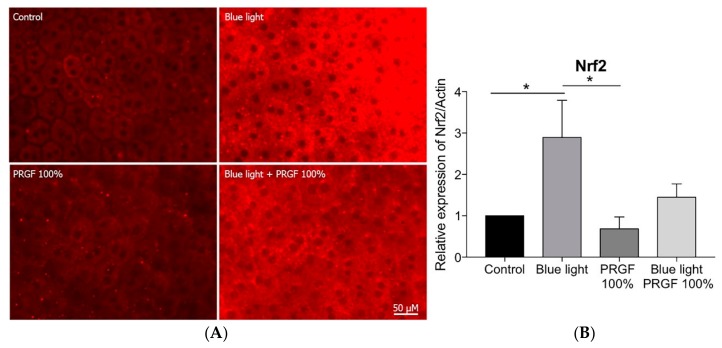
(**A**) RPE immunocytochemistry for Nrf2 (red) (*n* = 4), results showed that blue light increased Nrf2 staining, PRGF 100% combined with blue light resulted in reduced intensity of staining compared to blue light results; (**B**) Nrf2 gene expression was stimulated by blue light while PRGF 100% reduced it. Statistical analysis: One-way ANOVA, Tukey multiple comparison test, and * *p* < 0.05. Scale = 0.5 µM.

Using immunocytochemistry, we analyzed the expression of HO-1 (Figure 10A) and Nrf2 (Figure 11A). HO-1 expression was enhanced by the presence of blue light and reduced by PRGF 100% (Figure 10A).

Nrf2 expression was stimulated by the presence of blue light, but PRGF 100% combined with blue light reduced it (Figure 11A). In order to confirm the results obtained by immunocytochemistry, we performed qPCR on the same genes. The results showed that HO-1 expression was significantly increased by blue light, whereas PRGF, both on its own and in combination with blue light, reduced it as compared with the control levels (Figure 10B). Furthermore, Nrf2 expression was also significantly increased under blue light conditions as compared with the control and PRGF 100% reduced its expression although this was not statistically significant (Figure 11B).

## 3. Discussion

AMD is one of the leading causes of blindness across the world. This pathology affects the RPE, which is the layer of cells that provides nutrients to photoreceptors and protects them against the action of free radicals [41,42,43,44]. There are a number of factors that aggravate this disease and one of them is blue light, which is found in white light LEDs and several devices, meaning that in developed countries people are frequently exposed to it. It is known that pathological tissues are more sensitive to this harm because of their vulnerability which increases the risk of suffering from neurodegenerative issues such as AMD [45,46].

Blue light is known to increase the production of free radicals (ROS), which affect mitochondrial function by degrading the enzymes that participate in the electron transport chain. This can lead to a deficiency in ATP production and finally cell death [47,48,49,50].

There is some evidence showing that PRGF can act as a protector. Several authors have demonstrated the benefits of using PRGF for wound healing and inflammation in ocular surface pathologies [28,32,33,35,36,51,52]. This research group, therefore, wanted to test whether it could act as a neuroprotective agent on RPE cells.

To do this, we used ARPE19 cells, an immortalized cell line of RPE human cells. First, a viability test was performed in order to study how blue light and PRGF could affect the cells. Our results showed that blue light reduced cell viability by about 20% as compared with the control, although viability for the PRGF 10% and the blue light group demonstrated normal levels as compared with the control and PRGF alone was not found to be toxic for cells since viability levels were similar to that of the control.

Mitochondrial status results showed that mitochondrial activity was affected by blue light but that the presence of PRGF during exposure to blue light or PRGF exposure alone did not cause a decrease in mitochondrial function. The DHE results showed that PRGF reduces the presence of ROS when cells are exposed to blue light. This suggests that PRGF could protect mitochondria against the harmful action of blue light.

In order to test the antioxidant pathway of Keap1-Nrf2, we decided to study its main components. Keap1 acts as a modulator of Nrf2 expression, binding to Nrf2 in the cytoplasm and keeping it inactive. When ROS levels increase, Keap1 releases Nrf2, which translocates to the nucleus and activates the expression of other antioxidant molecules such as GCL, HO-1, and NQO1 [53,54]. To examine this, we quantified glutathione (GSH) concentration. GSH is one of the major antioxidant molecules released in order to protect cells against ROS insult [40,55,56,57,58,59]. Our results showed that blue light reduced the concentration of GSH. This is because GSH donates a proton to ROS to stabilize them, and thus transforms into its oxidized form (GSSG). However, PRGF, whether combined or not with blue light, increased GSH concentration back up to control levels. We also studied GCL, which is the enzyme involved in the first step of glutathione synthesis. GCL is formed by two subunits, GCLM (modulator subunit) and GCLC (catalyser subunit). We studied the molecular and protein expression of both subunits and the qPCR results showed that the gene expression of both increased after exposure to blue light. This makes sense because GSH production depends on GCL genes expression. In terms of proteins, our results showed that PRGF increased the expression of both subunits. As happened in the GSH quantification, the more ROS are released the more GSH is consumed, and therefore enzyme too. We also tested the gene expression of GSTP1, which is involved in modulating the donation to GSH of hydrophobic and electrophilic compounds in order to protect against ROS [60]. The molecular results showed that blue light increased the expression of this molecule and PRGF neutralized it.

The results of the molecular expression of Keap1 showed that blue light increased its expression. However, PRGF combined with the insult did not significantly reduced it. Nrf2 gene expression was also increased by the presence of blue light but reduced by PRGF. In order to test protein expression, we used a Western blot analysis of both cytoplasmic and nuclear Nrf2 fractions. The results showed that cytoplasmic Nrf2 expression did not change as a function of the treatment applied. However, the nuclear expression of Nrf2 increased in the presence of PRGF. This result, thus, demonstrates that PRGF stimulates the antioxidant pathway.

We also studied the expression of HO-1 and NQO1. Gene expression of HO-1 was shown to be significantly increased by blue light as compared with the control. However, PRGF in the presence of blue light led to an even greater increase. This result could be explained by the increase of Nrf2 in nucleus. In terms of protein expression, HO-1 cytoplasmic expression is also elevated under blue light conditions, and it is also significantly increased when PRGF is present as well as blue light. These results concurred with those of the immunocytochemistry.

NQO1 gene expression was also increased by the presence of blue light, although in this case, PRGF whether combined or not did not increase its expression compared to control.

The ex vivo experiment results showed a different response. The RPE cells from rat eyecups were exposed to different treatments (only for 3 h to avoid tissue deterioration). The response of the tissue was analyzed (gene expression and immunocytochemistry of HO-1 and Nrf2) to examine the effects of the action of PRGF and blue light. The results showed that HO-1 expression in both cases was slightly stimulated by the presence of blue light. However, in this case, PRGF reduced expression of both genes as compared with the control. Some research has shown that HO-1 expression differs in terms of the species involved. We also consider that HO-1 expression is time dependant, and that 3 h would not have been enough time for HO-1 to be expressed, as happened with cell culture experiments [61,62]. The Nrf2 results showed that its expression was stimulated by blue light and reduced by PRGF, following the same pattern as HO-1. This could be related to the species as well as to exposure time. Nevertheless, tissue integrity was better maintained when blue light was combined with PRGF.

In summary, blue light has been shown to alter the expression of different molecules in both ARPE19 cells and in RPE from rat eyecups. The expression of antioxidant genes such as Nrf2, HO-1, GCL, and NQO1 was increased in the presence of blue light. However, the results also demonstrated that PRGF blunts this effect by protecting cells against oxidative stress.

Future research work is needed to deepen our understanding of how PRGF can be used as a neuroprotector for retinal disorders.

## 4. Animals, Material, and Methods

### 4.1. PRGF

In accordance with the Helsinki Declaration of 2013, blood from 4 different healthy donors (all women, mean age 33 ± 7 years) was collected and placed in 9 mL tubes with 3.8% sodium citrate (Vacuette tube, Greiner Bio-One, Kremsmünster, Austria). The blood was then centrifuged at room temperature (Endoret System, BTI Biotechnology Institute, S.L., Vitoria, Spain). Whole plasma was collected after centrifugation, avoiding the leukocyte layer, and transferred to a 15 mL tube. Plasma was mixed with calcium chloride for fibrinogen activation and incubated for 30 min at 37 °C, or until clotting was achieved. The supernatant was collected and exposed to heat (56 °C) for 1 h in order to inactivate the complement system. After that, the plasma was filtered, aliquoted, and kept at −4 °C until use (less than 6 months).

### 4.2. Cell Culture Analysis

Human ARPE19 cells (ATCC, Wesel, Germany) were grown in a culture medium that consisted of DMEM-F12 solution (Sigma-Aldrich, St Louis, MO, USA), supplemented with 2% antibiotic penicillin/streptomycin (Sigma-Aldrich, St Louis, MO, USA)) and 10% FBS, and kept in a humidified atmosphere of 5% CO_2_, at 37 °C. Doubling growth time was approximately 60 h. Either 100 µL or 2 mL of cell culture (approximately 10 × 10^4^ cells/mL) were taken and placed in 96-well plates or T75 flasks, respectively.

After allowing the cells to settle (approximately 24 h in the case of the 96-well plates and 72 h in that of the T75 flasks) the samples were subjected to the treatment regimens indicated in Table 1.

Blue light LEDs (Electro DH SL, Barcelona, Spain) were used to deliver light (465–475 nm, 400 lux, 18 W/m^2^) to the cultures and the temperature was monitored to maintain it at 37 °C. 

The aim of giving cells a one-hour pretreatment in the dark was to let them settle in the new culture medium.

### 4.3. Cell Viability

Cell viability was assessed by the MTT reduction assay. Briefly, cells were subjected to the appropriate treatment and then MTT (Sigma-Aldrich, St Louis, MO, USA)) was added at a final concentration of 0.5 mg/mL for 75 min at 37 °C. After that, the medium was removed and the reduced MTT (blue formazan crystals) was solubilized by adding 100 µL DMSO to each well. After agitation of the plates for 5 min, the optical density of the solubilized crystals was measured using an automated microplate reader at a wavelength of 570 nm (PerkinElmer 2030 Multilabel Reader, Victor X5, Waltham, MA, USA).

### 4.4. JC-1 and DHE

For the analysis of mitochondrial status, cells were incubated with JC-1 (Sigma-Aldrich, St Louis, MO, USA) dye (1.5 µg/mL) for 30 min. The accumulation of JC-1 in mitochondria appears as a red/orange fluorescence (590 nm) in healthy organelles and green when it is depolarised (530 nm). Fluorescence images of cells were recorded and the relative levels of the intensities of green/red JC-1 fluorescence quantified using a microplate reader at wavelengths of 570 nm and 535 nm.

For the analysis of ROS, culture medium was removed and the cells incubated with DHE (Thermo Fisher Scientific, Waltham, MA, USA) (40 µM), for 30 min after which they were washed twice with fresh medium. Images of the cultures were immediately recorded using phase fluorescence/contrast microscopy. ROS formation was determined by measurement of the ratio fluorescence at 370 to 420 nm (for cytoplasm of living cells) and 535 to 610 nm (for chromatin of living cells, in red) using a microplate reader.

### 4.5. GSH Quantification

A glutathione assay kit (703002, Cayman Chemical, Ann Arbor, MI, USA) was used to quantify GSH concentration. Briefly, ARPE19 cells were centrifuged after collection. The resulting pellet was homogenized and sonicated for 1 min in 500 µL of cold buffer. After this, the cells were centrifuged again at 10,000× *g* 15 min at 4 °C. The supernatant was mixed with 500 µL of MPA reagent and mixed by vortexing, left to stand at room temperature for 5 min and, then, recentrifuged at 3000× *g* 4 min. Then, 50 µL of TEAM Reagent was added per ml of sample and vortexed. Finally, 50 µL of sample and 150 µL of Assay Cocktail were added to each well of a 96-well plate. GSH levels were tested at five minutes intervals for 30 min at 405–414 nm in a plate reader.

### 4.6. Immunocytochemistry

ARPE19 cell cultures were fixed in cold methanol or 4% paraformaldehyde for 10 min which was followed by washing in phosphate buffer (PBS). After incubation in goat serum (10% in PBS) for 60 min and washing in PBS, cultures were then exposed overnight at 4 °C to anti-HO-1 (Enzo LS, Farmingdale, NY, USA, 1:100). After washing with PBS, cultures were, then, exposed for 2 h to the appropriate secondary antibody conjugated either to Alexa Fluor 488 or to Alexa Fluor 594 (1:300), and then washed in buffer. After that, DAPI (0.2 µg/mL) was added to a wash solution. Images were obtained using a Leica DMI6000B fluorescence microscope (Leica Microsystems, Wetzlar, Germany).

### 4.7. Western Blot Analysis

ARPE19 cells were collected by scraping them from the T75 flasks, followed by centrifugation and resuspension in a cocktail lysis buffer that contained phosphatase and protease inhibitors (Sigma, Aldrich). After freezing and thawing in combination with sonication, the supernatant with its protein content was collected. In order to extract the nuclear protein fraction, the pellet was exposed to a further extraction process with a buffer (HEPES, MgCl2, NaCl, EDTA, glycerol, and DTT). Defined amounts of protein and sample buffer (2 M Tris/ HCl, pH 6.8, containing 8% SDS, 40% glycerol, 8% mercaptoethanol, and 0.002% bromophenol blue) were then mixed together and immediately heated for 5 min at 95 °C. Equal amounts of proteins were fractionated by electrophoresis using 10% polyacrylamide gels containing 0.1% SDS. Proteins were transferred to 0.22 µm nitrocellulose membranes and were incubated overnight at 4°C with one of the following primary antibodies: anti-actin (MAB1501, Millipore, Burlington, MA, USA, 1:4.000), anti-lamin A/C (Santa Cruz Biotechnology, Dallas, Texas, USA, 1:100), anti-Nrf2 (Abcam, Cambridge, UK, 1:1.000), Anti-HO-1 (ADI-SPA895, Enzo LS, 1:200), GCLC (Thermo Fischer, Waltham, MA, USA, 1 µg/mL), GCLM (Thermo Fischer, Waltham, MA, USA, 1:1.000). Detection was then performed with appropriate biotinylated secondary antibodies. The final nitrocellulose blots were developed with a 0.016% *w*/*v* solution of 3-amino-9-ethylcarbazole in 50 mM sodium acetate (pH 5.0) containing 0.05% (*v*/*v*) Tween-20 and 0.03% (*v*/*v*) H_2_O_2_. The colour colorimetric reaction was stopped with 0.05% sodium azide/PBST solution and the density of the individual bands quantified using IMAGEJ Software (U.S. National Institutes of Health, Bethesda, MD, USA).

### 4.8. RNA Extraction and mRNA Analysis

Total RNA from ARPE19 cells was extracted using the Illustra RNAspin Mini kit from GE Healthcare. The purity of the RNA was then checked by the A260/A280 and A260/A230 ratio. Next, 0.5 µg of total RNA was used for linear conversion of RNA to cDNA with High Capacity RNA-to-cDNA Master Mix (Cat. Num. 4387406, Applied Biosystems, Waltham, MA, USA) following the manufacturer’s instructions (60 min at 37 °C, 5 min at 95 °C, and held at 4 °C). Primers (see Table 3) were customised using PrimerBLAST and synthesized by Sigma-Aldrich. Gene expression was performed by relative quantification in a 7500 Real-Time PCR System (Applied Biosystems, Waltham, MA, USA) using a Power SYBR Green PCR Master Mix (Cat. Num. 4367659, Applied Biosystems, Waltham, MA, USA) and the ∆∆Ct method. Each sample was analyzed in triplicate for each of the experiments (*n* = 4). Data were analyzed using SDS 1.4 software (Applied Biosystems, Waltham, MA, USA).

### 4.9. Animals

This study was performed in accordance with the ARVO Statement for the Use of Animals in Ophthalmic and Vision Research. The procedures and experimental designs were approved by the Animal Experimentation Ethics Committee of the University of Oviedo (Oviedo, Principado de Asturias, Spain) (PROAE 17/2017) and complied with European and national laws. 

Wistar male rats of about 500 gr (*n* = 40) were anaesthetised with ketamine/xylazine 80/10 mg/kg. A second injection of 1/3 of the initial anaesthetic solution was used to keep the rats asleep for 60 min. After that, they were euthanised with a penthobarbithal injection and their eyes enucleated. After this, the cornea, lens, and vitreous were removed and the eyecups were placed in a 96-well plate. Blue light LEDs were used to deliver light to the RPE at 465–475 nm (900 lux, 32 W/m^2^) for 3 h.

#### 4.9.1. Immunocytochemistry

Eyecups of 16 male Wistar rats were fixed in cold methanol for 60 min, then, washed in phosphate buffer (PBS). After incubation in goat serum (10% in PBS) for 60 min and washing in PBS, the cultures were then exposed overnight at 4 °C to anti-ZO-1 (Thermo Fischer, Waltham, MA, USA, 1:100), anti-HO-1 (Enzo LS, Farmingdale, NY, USA, 1:100), and anti-Nrf2 (Abcam, Cambridge, UK, 1:100). After washing with PBS, cultures were then exposed for 2 h to the appropriate secondary antibody conjugated to either Alexa Fluor 488 or Alexa Fluor 594 (1:300) and washed in buffer. After that, DAPI (0.2 µg/mL) was added to a wash solution. Images were obtained using a Leica DMI6000B fluorescence microscope (Leica Microsystems, Wetzlar, Germany).

#### 4.9.2. RNA Extraction and mRNA Analysis

RPE from male Wistar rats (*n* = 24) was removed from the eyecup by scraping carefully with a scalper. Total RNA from RPE was extracted using the Arcturus PicoPure RNA Isolation Kit (Applied biosystems, Thermo Fischer, Waltham, MA, USA). The purity of RNA was checked by the A260/A280 and A260/A230 ratio. Next, 0.5 µg of total RNA was used for the linear conversion of RNA to cDNA with High Capacity RNA-to-cDNA Master Mix (Cat. Num. 4387406, Applied Biosystems, Waltham, MA, USA) following the manufacturer’s instructions (60 min at 37 °C, 5 min at 95 °C, and held at 4 °C). Primers (see Table 4) were customised using PrimerBLAST and synthesized by Sigma-Aldrich. Gene expression was analyzed by relative quantification (∆∆Ct method) on a 7500 Real-Time PCR System (Applied Biosystems, Waltham, MA, USA) using a Power SYBR Green PCR Master Mix (cat. no. 4367659, Applied Biosystems, Waltham, MA, USA). Each sample was analyzed in triplicate for each of the experiments (*n* = 4). Data were analyzed using SDS 1.4 software (Applied Biosystems, Waltham, MA, USA).

### 4.10. Statistical Analysis

All statistical tests were analyzed using Graphpad Prism version 7.0a for Mac (GraphPad Software, La Jolla, CA, USA). To assess the statistical significance of two mean differences, we used a one-way ANOVA test. For the statistical comparison of mean differences between treatments we used a Tukey multiple comparison test. Differences were considered statistically significant when *p*-values were < 0.05.

## Figures and Tables

**Table 1:** Experimental groups for ARPE19 analysis.

Treatments	Medium	Dark/Blue Light
Control	DMEM F12 + FBS 1%	Dark 19 h
Blue light	DMEM F12 + FBS 1%	Dark 1 h + blue light 18 h
PRGF 10%	PRGF 10% on culture medium DMEM F12 +FBS 1%	Dark 19 h
PRGF 10% + blue light	PRGF 10% on culture medium DMEM F12 +FBS 1%	Dark 1 h + blue light 18 h

**Table 2:** Experimental groups for ex vivo model.

Treatments	Medium	Dark/Blue Light
Control	DMEM F12 + FBS 10%	Dark 3 h
Blue light	DMEM F12 + FBS 10%	Blue light 3 h
PRGF 100%	PRGF 100%	Dark 3 h
PRGF 100% + blue light	PRGF 100%	Blue light 3 h

**Table 3:** Sequences of human primers used.

Gene	ID	Forward	Reverse
*Actin*	NM_001101.4	5′-ATTCCAAATATGAGATGCGTTGTT-3′	5′-GTGGACTTGGGAGAGGACTG-3′
*NQO1*	NM_000903.3	5′-TTGAGCGAGTGTTCATAGGAGAG-3′	5′-CCTTCTTACTCCGGAAGGGT-3′
*HO-1*	NM_002133.2	5′-CTGGAGGAGGAGATTGAGCG-3′	5′-ATGGCTGGTGTGTAGGGGAT-3′
*GCLM*	NM_002061.3	5′-AGCAXTTTCTCGGCTACGATT-3′	5′-GCGGGAGAGCTGATTCCAAA-3′
*GCLC*	NM_001498.3	5′-TGGAGACCAGAGTATGGGAGT-3′	5′-AAGGTACTGAAGCGAGGGT-3′
*GSTP1*	NM_000852.3	5′-AGGCCTTCGCTGGAGTTTC-3′	5′-CGGCCTCGAACTGGGAAATA-3′
*Keap1*	NM_012289.3	5′-CCATGAAGCACCGGCGAAGTGCC-3′	5′-GTCTGTATCTGGGTCGTAACACTCCAC-3′
*Nrf2*	NM_001313904.1	5′-TCAGTCAGCGACGGAAAGAG-3′	5′-GTGGGCAACCTGTCTCTTCAT-3′

**Table 4:** Sequences of rat primers used.

Gene	ID	Forward	Reverse
*Actin*	NM_031144.3	5′-GCGTCCACCCGCGAGTACAAC-3′	5′-CGACGACGAGCGCAGCGATA-3′
*HO-1*	NM_012580.2	5′-CAGCCCCAAATCCTGCAACAGA-3′	5′-CAACATGGACGCGCCGACTACCAA-3′
*Nrf2*	NM_001145412.3	5′-ATTTGTAGATGACCATGAGTCGC-3′	5′-TCCTGCCAAACTTGCTCCAT-3′

## Data Availability

All the obtained data used to support the findings of this study are available from the corresponding author upon request.
